# Personal and environmental factors to consider when aiming to improve participation in physical activity in children with Spina Bifida: a qualitative study

**DOI:** 10.1186/s12883-015-0265-9

**Published:** 2015-02-10

**Authors:** Manon AT Bloemen, Olaf Verschuren, Claudia van Mechelen, Hanneke E Borst, Arina J de Leeuw, Marsha van der Hoef, Janke F de Groot

**Affiliations:** Research Group Lifestyle and Health, HU University of Applied Sciences Utrecht, 101, 3584 CJ Utrecht, The Netherlands; Child Development and Exercise Center, Wilhelmina Children’s Hospital, University Medical Center Utrecht, Lundlaan 6, 3584 EA Utrecht, The Netherlands; Brain Center Rudolf Magnus and Center of Excellence for Rehabilitation Medicine, University Medical Center Utrecht and De Hoogstraat Rehabilitation, Rembrandtkade 10, 3583TM Utrecht, The Netherlands

**Keywords:** Youth, Spina bifida, Facilitators, Barriers, Physical activity

## Abstract

**Background:**

Youth with spina bifida (SB) are less fit and active than other groups with childhood disability. While recent studies have shown benefits of exercise training, the increased fitness levels do not sustain or lead to increased levels of physical activity (PA) in these children. Therefore, it seems important to explore which factors are associated with participation in PA (or lack of) in youth with SB. The objective of this study is to describe both personal and environmental factors that are important for participation in physical activity as experienced by these children and their parents, in order to better develop intervention strategies to improve participation in PA in youth with SB.

**Methods:**

Eleven semi-structured interviews with parents of children with SB aged 4–7 years, nine focus groups with youth with SB (n = 33, age 8–18 years) and eight focus groups with their parents (n = 31) were conducted, recorded and transcribed verbatim. Two independent researchers analyzed the data. Central themes for physical activity were constructed, using the model for Physical Activity for Persons with a Disability (PAD model) as a background scheme.

**Results:**

Data showed that youth with SB encountered both personal and environmental factors associated with participation in PA on all levels of the PAD model. Bowel and bladder care, competence in skills, sufficient fitness, medical events and self-efficacy were important personal factors. Environmental factors that were associated with physical activity included the contact with and support from other people, the use of assistive devices for mobility and care, adequate information regarding possibilities for adapted sports and accessibility of playgrounds and sports facilities.

**Conclusions:**

Our findings suggest that a variety of both personal and environmental factors were either positively or negatively associated with participation in PA. An individual approach, assessing possibilities rather than overcoming barriers within and surrounding the child may be a good starting point when setting up intervention programs to improve participation in PA. Therefore, assessment of both personal and environmental factors associated with physical activity should be standard care within multidisciplinary intervention programs aimed to encourage healthy active lifestyles in youth with SB.

## Background

Spina Bifida (SB) is the most frequently seen congenital deformity of the neural tube. The incidence ranges from 3–4 to 7–12.8 new cases per 10,000 births [1]. The malformation of the spinal cord and often the brain can result in both motor and sensory impairment, incontinence for bowel and bladder and cognitive impairment [2]. Due to advances in the medical approach, mortality rates have decreased over the last years and 75%-80% of children with SB can now be expected to live to be adults [3,4]. This requires a different approach in management of these patients from childhood into adulthood, not only focusing on the pathological aspects, but also at the secondary prevention and healthy active living [5]. In optimizing health outcomes of youth with SB, like in other youth with chronic childhood conditions, physical activity (PA) is an increasingly important factor to consider. Not only because of its presumed relation with fitness and health [6], but also because of increasing evidence suggesting that healthy and active children become healthy and active adults [7].

The risk for reduced levels of activity and fitness has been confirmed in a recent study in ambulatory children with SB [8]. Additionally, in a study with adolescents and young adults with SB, 39% were classified as inactive, with 37% as extremely inactive. The average aerobic capacity was 42% lower than their typically developing peers, with obesity found in 35% [9]. Even more, youth with SB not only have low fitness and PA compared to their typically developing peers; they are also in the lowest range of fitness and PA when compared to other children and adolescents with physical disabilities [10,11]. The increased risk for components of metabolic syndrome due to the low PA has been described for youth, adolescents and young adults with SB [12,13].

Training programs in children and adolescents with disabilities, including SB, have shown positive results in fitness [14-17]. At the same time, these studies have shown that the benefits of exercise training, e.g. the increased fitness levels do not sustain or lead to increased levels of physical activity in youth with SB, Given the benefits of PA in maintaining these gains in fitness and other health benefits, it seems important to explore which personal and environmental factors are associated with participation in PA (or lack off) in this specific population.

A recent review describes factors for PA in youth with a disability [18]. The authors concluded that most available literature included several types of disabilities, making it difficult to understand whether the factors would differ between specific diagnoses [18]. Given the fact that youth with SB are even less active than other groups with childhood disability, it is important to see if there are factors that are specific for participation in PA (or lack off) in youth with SB. Shields et al. also stated that further research should not only examine negative factors or barriers but should also focus on positive factors or facilitators for PA, as these positive factors might be successful strategies to use in the development of interventions aimed at increasing participation in PA [18]. To date, no study has explored the specific factors for participation in PA in youth with SB. Knowing that parents play an important role in the lives of their children it is imperative to consider their perspectives as well on their child’s potential to be physically active [19,20]. Therefore, the objective of this study is to describe both personal and environmental factors that are important for participation in PA as experienced by both youth with SB and their parents, in order to better develop intervention strategies to improve participation in PA in this population.

## Methods

### Design and data collection

This study employed a descriptive qualitative design, with a thematic analysis [21]. Social constructivism was the base for this research, as referred to by Cresswell et al. as being a “subjective meaning” of how people experience their world. In this approach questions remain broad to encourage the participants to construct their meaning, which is supported by interaction with others (the social part of social constructivism) [21,22]. A recent paper about qualitative data collection with children indicated that children are able to talk about and share their experiences and views [23]. Therefore, the data was collected using focus groups with children and adolescents 8 – 18 years of age and their parents. Separate focus groups for the children and adolescents and the parents were conducted, so that possible differences between these groups could become evident. Focus groups were chosen since the interaction during focus groups can be beneficial, as the members are encouraged to share and clarify individual and their shared ideas and opinions [24]. A number of six to ten participants is found ideal to create sufficient interaction between the participants with different views and experiences [25,26]. In this study however, the number of participants per focus group was decreased to three to six because of the frequent attention difficulties typical in youth with SB [27,28]. Because younger children (4–7 years of age) were not considered capable to specify factors associated with participation in PA, due to logistic reasons, semi-structured interviews and not focus groups were conducted with one or both parents within this group. For this study PA consists of both PA in activities of daily life, such as (hand) biking to school or active play, and participation in (un)organized sports. It is defined as “any bodily movement, produced by skeletal muscles, that results in energy expenditure” [29].

Both the focus groups and the individual interviews were conducted in a rehabilitation center, a pediatric physical therapy institution or in the home situation. Experienced and trained interviewers with a (pediatric) physical therapy background conducted the interviews. Open-ended questions (see Appendix 1) were used to allow participants to express their feelings and opinions in their own words, where clarification could be provided when necessary. Participants were not directed towards any particular pre-conceived response. All focus groups and interviews were audio taped and filmed.

Prior to the focus groups and interviews general information regarding family composition, education level, ambulatory classification and PA patterns was gathered using a standardized questionnaire.

The study was approved by the Internal Review Board from the HU University of Applied Sciences Utrecht.

### Participants

In order to include the whole range of elementary and secondary school up to young adulthood, this study focused on children and adolescents with SB 4 – 18 years of age and their parents. A purposeful, maximum variation sampling was used so all possible factors would emerge [30]. The participants were recruited through the BOSK (Association from and by parents from children, adolescents and adults with a disability), pediatric physical therapists and several SB outpatient services in the Netherlands. In order to reduce the burden of travel and time, the groups were formed by convenience, rather than stratification by age, gender or level of PA. Youth with SB and/or their parents were included if they were 8 – 18 years of age or had children with SB aged 4–18 years. Written informed consent was signed by youth 12 years of age and older as well as by their parents prior to taking part in this research. For children < 12 years of age, only parents signed informed consent in line with Dutch law. The children, adolescents and the parents were excluded if they were insufficient in the Dutch language or if participation was not possible due to cognitive or behavioral problems.

### Data analysis

All focus groups and interviews were transcribed verbatim based on the audio- and videotapes and transcriptions were checked (by CvM) independently to enhance dependability. After this step text that was determined as not relevant (such as “hhmmm”, “aha”) was deleted after consensus [25]. A thematic analysis was performed with an inductive strategy [21]. It was an iterative process in which fragments were coded, resulting in subthemes and finally themes were determined for every interview and focus group. Step one consisted of defining a text section as a PA, a positive or a negative determinant or a solution. Positive and negative determinants were aspects that were already present, whereas a solution was defined as an aspect that was not yet experienced in real life by the participants of that focus group or interview. During step two, the text was classified as a personal or an environmental determinant using the International Classification of Functioning, Disability and Health for Children and Youth (ICF-CY). The third step specified the detailed description of the PA, positive or negative determinant or solution.

The analyses for each focusgroup/interview were performed by two independent researchers with varying experiences in working with children and adolescents with SB (0 – 15 years). Consensus was reached after every step. In case of no consensus, a third researcher was consulted who had extensive experience in research in children and adolescents with SB. After analyzing all focus groups and interviews separately, central themes were constructed by two independent researchers (CvM, MB). The solutions from the separate focus groups and interviews were compared to the positive determinants; if a solution was already mentioned as a positive determinant in another focus group or interview, it was specified as a positive determinant theme. After construction of these central themes, they were discussed with the third researcher (JdG) and several experts working in the field of pediatric medicine. Member checking was performed by presenting the central themes to a different group of parents of children and adolescents with SB, asking if they agreed with the results and if there were any missing determinants. The final step consisted of categorizing the central themes in modifiable determinants, partly modifiable determinants and non-modifiable determinants by the two independent researchers (CvM, MB).

The Physical Activity for persons with Disability model (PAD model) was used as a background scheme [31]. In many studies looking at factors associated with participation in PA, the PAD model is being used to identify emerging themes [18,32]. This model combines the ICF with the model of Attitude, Social Influence and Self-Efficacy (ASE model) [31]. This results in a model, enlarging the personal and environmental factors as part of the ICF model that either facilitate or hinder the intention to participate in physical activity. The personal factors consist of the levels of *“Intention”*, *“Attitude”*, *“Self-efficacy”*, *“Health condition”* and *“Facilitators and Barriers”. “Intention”* is the central determinant for participation in PA within the PAD model. Without intention to be active, a person is most likely not going to be active. At the same time though, a person may very well have the intention to be active, but this intention is influenced for better or worse by other contextual factors both at the personal and environmental levels [31]. *“Attitude”* is defined as what an individual thinks and expresses about an active lifestyle for him- or herself and *“Self-Efficacy”* is the confidence that an individual has for performing PA. *“Health condition”* refers to specific aspects related with the diagnosis, in this case SB. The environmental factors only consist of the level of *“Social Influence”*, defined as what another person thinks about PA for that individual, and, like the personal factors, the level of *“Facilitators and Barriers”* [31].

To enhance the credibility and conformability, two independent researchers with varying experiences in pediatric physical therapy performed the analyses. In case of no consensus, a third researcher was consulted who had extensive experience in research in children and adolescents with SB [33,34]. Several experts working in pediatric medicine performed skeptical peer review to ensure dependability [33,34]. In addition member checking was performed by presenting the results to a different group of parents of children and adolescents with SB, leading to credibility [33,34].

The data was analyzed through MaxQDA version 10 (VERBI, Berlin, Germany) to enhance standardization and transparency [35].

## Results

Eleven semi-structured interviews with 13 parents from young children with SB, nine focus groups with youth (n = 33) with SB and eight focus groups with their parents (n = 31) were conducted. Participants did not discuss any new factors after the 7^th^ focus group and 10^th^ interview. Therefore, the researchers were confident that informational saturation was achieved. The children and adolescents attended both regular schools and schools for special education and their mobility varied from normal ambulatory to non-ambulatory [36]. Table 1 provides an overview of the characteristics of both the children and adolescents and the parents. In the Netherlands, children with special needs often attend special education schools, which are regionally distributed and are funded, like regular schools, by the Dutch government.

**Table 1:** **Characteristics of the children, adolescents and their parents**

		**Parents (n = 13: from children 4–7 years)**	**Parents (n = 31: from children and adolescents 8–18 years)**
**Age**		Mean 39 years (range 27–44)	Mean 47 years (range 34–64)
**Sex (F/M)**		11/2	25/6
**Number (%) adhering to the Dutch guidelines for healthy PA**		12 (92 %)	26 (84 %)
**Level of education (%) university or professional level)**		35	30
		**Children 4–7 years (n = 11)**	**Children and adolescents 8–18 years (n = 33)**
**Age**		Mean 6 years (range 4–7)	Mean 13 years (range 8–18)
**Sex (F/M)**		4/7	15/18
**Diagnoses**		9 SB 2 SB with hydrocephalus	26 SB 7 SB with hydrocephalus
**Mobility (Hoffer classification** [34]**)**			
	**Normal ambulatory**	1	2
	**Community ambulatory**	2	6
	**Household ambulatory**	1	5
	**Non ambulatory**	7	20
**Number (%) adhering to the dutch guidelines for healthy PA**		6 (55 %)	17 (57 %)
**Number (%) with siblings**		9 (90%)	28 (85%)
**Education (regular/special)**		6/5	9/24

SB = spina bifida, PA = physical activity, F = Female, M = Male.

**Figure 1:**
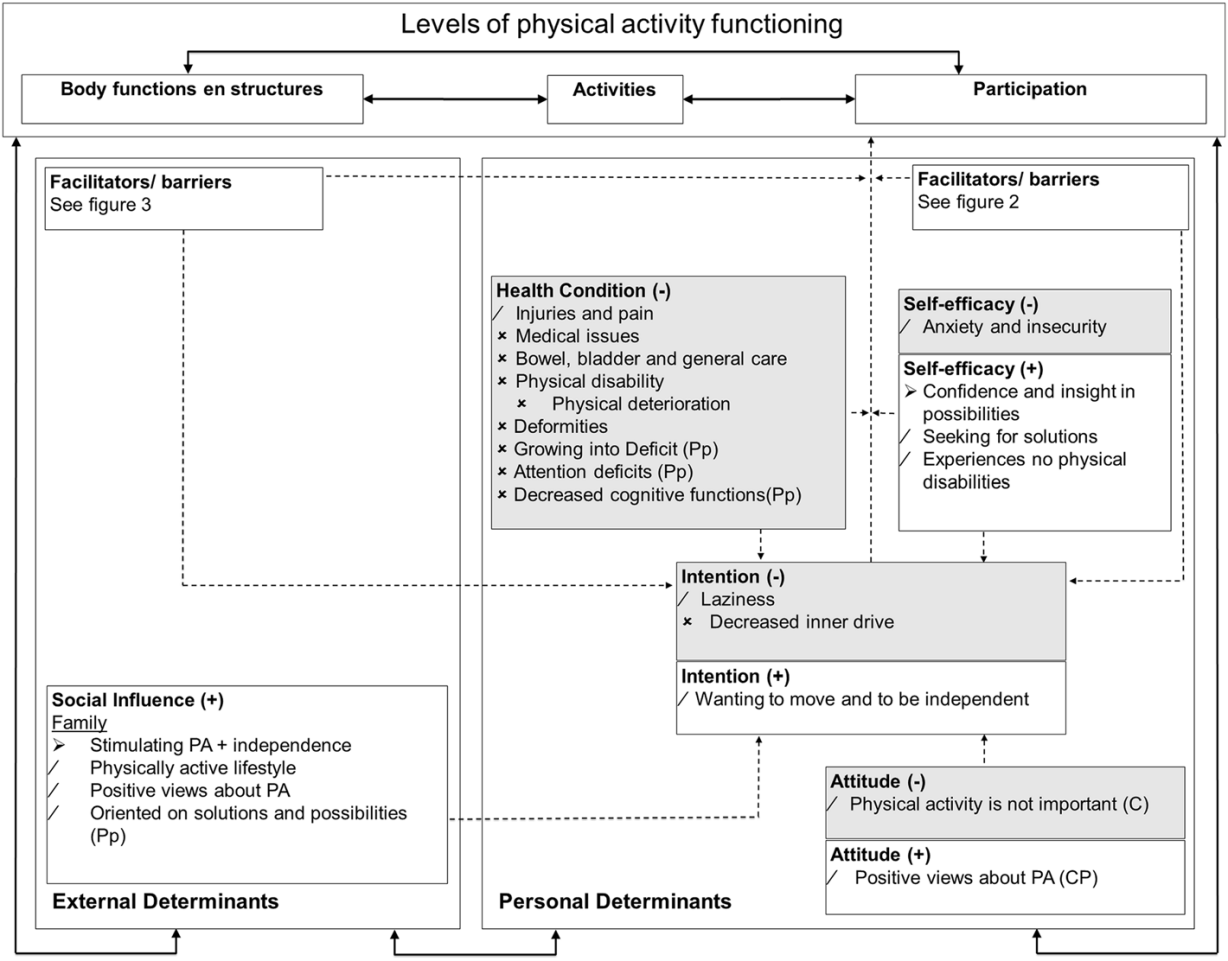
**Results of this study incorporated in the PAD model [** 31 **].** ➢ Modifiable factors. / Partly modifiable factors. × Not modifiable factors. P Parents (8–18 year old children). p parents (4–7 year old children). C Children and adolescents (8–18 years). When there is no indicator behind a factor, it means the factor is mentioned in (P), (p) and (C). PA Physical Activity. Red Negative factors. Green Positive factors.

**Figure 2:**
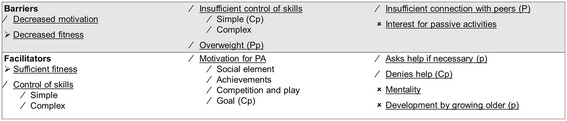
**Personal factors, barriers and facilitators.** ➢ Modifiable factors. / Partly modifiable factors. × Not modifiable factors. P Parents (8–18 year old children). p parents (4–7 year old children). C Children and adolescents (8–18 years). When there is no indicator behind a factor, it means the factor is mentioned in (P), (p) and (C). PA Physical Activity. Red Negative factors. Green Positive factors.

**Figure 3:**
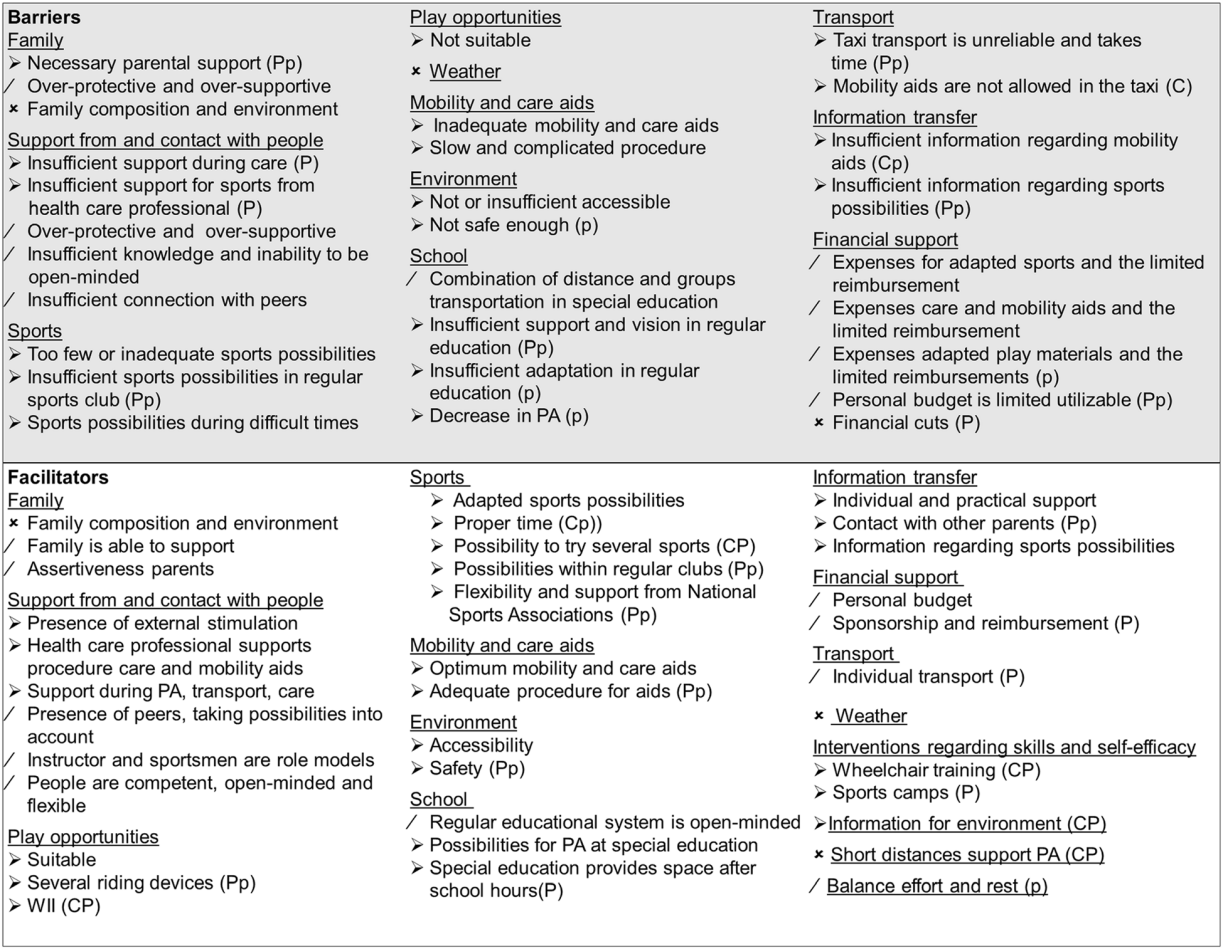
**Environmental factors, barriers and facilitators.** ➢ Modifiable factors. / Partly modifiable factors. Not modifiable factors. P Parents (8–18 year old children). p parents (4–7 year old children). C Children and adolescents (8–18 years). When there is no indicator behind a factor, it means the factor is mentioned in (P), (p) and (C). PA Physical Activity. Red Negative factors. Green Positive factors.

Data showed that youth with SB encountered a variety of both positive as negative personal and environmental factors for PA during childhood on all levels of the PAD model, with only minor differences between the children, adolescents and the parents. Individual differences were present and the factors varied in modifiability. Figures 1, 2 and 3 present overviews of the central themes on the different levels of the PAD model, the most important issues are discussed in the text. The quotes (P = Parents from children 8–18 years, p = parents from children 4–7 years, C = Children and adolescents 8–18 years) represent the literal translation of what the children, adolescents or parents said during the focus groups or the interviews.

### Personal factors

#### Intention

Wanting to be physically active and to be independent seems to be a very strong positive theme; *“(C) I always self propel my wheelchair,….at a certain point,… you have to do it yourself later on*”. However, there also seems to be a large group of children and adolescents who lack an inner drive for PA, which seems to be difficult or sometimes even impossible to change; *“(p) the complication is, that the stimulation always has to come from us….what I experience from my healthy children,…is they ask us for help,…. and say ‘now you have to help me because ….. I want to do this and that’, they ask. He doesn’t ask, it always has to be stimulated by us*”.

#### Attitude

Both the children, adolescents and the parents described a positive attitude towards PA in the children and adolescents, for example because of expected health benefits and social contacts; *“(C) if you play sports, you get energy….., you’ll become fit and yes you’ll notice”, “(C) because you’re around people, you make contact with people, sometimes you make friends”.* In the children and adolescents however, was also mentioned that PA was not important.

#### Self-efficacy

Both the children, adolescents and parents pointed out the importance of self-confidence; it seems to be crucial to have a notion and realization of own capacities and possibilities. Positive experiences were described of training programs or camps that also focus on developing self-efficacy. When this was explained by a girl *“(C) because I dare to do things now that others don’t dare”*, an adolescent reacted *“(C) yes, I would also like to do that, for my self-confidence because…if you fall, you know what to do”*, the girl reacted *“(C) yes, that’s what I mean”*.

#### Health condition

Medical problems, bowel and bladder care, injuries and pain, disabilities, deterioration and deformities were important physical negative factors of SB besides attention and cognitive dysfunctions. The bowel and bladder care influenced PA mostly when the child was incapable of self-catheterization*; “(P) really an obstacle…., every 3.5 hour it has to happen…. so you always have to plan ahead, or you have to go back and forth…., you always have to say ‘it’s not possible to come directly after school because he has to go to the toilet first’, it is even a bigger obstacle than the handicap, you always have to be there as a parent…”*. According to parents, both the physical and the cognitive dysfunctions may lead to growing into deficit when these children and adolescents grow older, meaning that the differences between the children and adolescents with SB and typically developing peers become more evident.

#### Facilitators and barriers within the child

The competence in both simple as complex skills are important facilitators and barriers; *“(P) Wheelchair training, that is very important I think, …..that they really learn to go up and down stairs….. ….she can do much more now….a lot of places are not adjusted for wheelchairs ….and you can just go….your life becomes a lot more fun”.* Social consequences are mentioned as an important facilitating motivational aspect. A requirement for PA seems to be a sufficient level of fitness, *“(C) being unfit”* was mentioned as a barrier because *“(C) you get tired more easily”*. Overweight or obesity is seen as a barrier for PA, because transfers are more difficult.

### Environmental factors

#### Social influence

The importance of stimulating the child in a physically active and independent lifestyle was emphasized upon. All parents believed that PA is healthy because of multiple reasons such as positive effects on health, social relations and general development. Additionally, the parents reported a solution-orientated approach within the family, as a positive factor; not emphasizing on the difficulties, but focusing on solutions and possibilities. “*(C) I think partly maybe the way I was brought up, because my parents they always say, you have to propel yourself as much as possible, because your fitness will increase……if I start to complain ‘I’m tired, I want to go home’, well, they ignore it……I think it is ok, I think I will benefit later on. On the other hand, at that moment when I’m tired and they don’t want to push me, I am mad (laughing)”*.

### Facilitators and Barriers within the environment

#### - Other people

A major theme was the support from and contact with people in general. The protective attitude towards children and adolescents with a disability such as SB, was mentioned as an important barrier, but also the inability to be open-minded and flexible. A girl said *“(C) sometimes I see handicapped children….older than I am, and they are treated like they are much younger and then I think, you just can’t do that”*, after which another boy remarked *“(C) well, I think it is like that, because they usually think that you’re also mentally handicapped and that’s why they think oh, he’s not that smart”*. One of the adolescents trained in a local fitness centre and she stated: *“(C)….they easily think that activities are too hard….if I for example say ‘I want to do this and that’ he will say ‘that is too hard for you…..what if something happens…’*….*well. It is difficult to say otherwise…*”. People in general seem to have a lack of knowledge about possibilities for PA in children with SB, but it varies widely how they cope with this. Certain people are willing and able to adjust activities, but also examples that were the opposite were mentioned. *“(P) We now have a teacher who absolutely doesn’t want to make adjustments in the physical education class. They just say ‘if he can’t do it, he can’t do it’. We had a huge discussion about his grade for physical education this year. She didn’t want to give a higher grade than a C, because, well, that was just not possible.”*

#### - Possibilities to participate in sports

Sports possibilities were another major theme. In general, there were not enough suitable sports possibilities and the possibilities of participating in regular, local sport clubs were scarce. If they have to travel to sports clubs further away, transport problems will arise and it will also be more time consuming. *“(C) It has to be in the neighborhood….so you can go by yourself…so your parents don’t have to take you. Yes, because when you grow older, it is annoying always having your parents around”*. A tendency towards more sport possibilities for children and adolescents with a disability in local and regular clubs is noted by the parents, for example a regular local soccer club that set up a team for children with physical disabilities” *(p) there are a lot of enthusiastic people who said it really fits in our club, and we’re going to take care of it!”*. The support from the national sports associations for participation in local and regular sports clubs seems to increase and the necessity of this support was underlined, *“(P) they have a huge roll and they think it is important”, “(P) you notice that they’re working on it, but I think it should go faster”.*

#### - Assistive devices

The importance of good assistive devices for optimal mobility and personal care was highlighted; *“(C) you can achieve the same things with an assistive device as an able bodied person…, a wheelchair is a replacement of your legs…..but then you need good equipment… I should not have to adjust to my equipment…it should exactly be the other way around”*. Even though all agreed upon the importance of optimal assistive devices, the reality is often otherwise unfortunately. *“(P) Since September, she can’t handbike anymore, ….if everything is ok again, the summer is probably over, that’s such a waist,…her friend lives 3 kilometer away, she can easily bike it, but now we have to take her,....she is very limited because of assistive mobility devices that do not work”.*

#### - Accessibility

The environment, such as playgrounds and sports facilities, lacks accessibility. Playgrounds with sand or grass are difficult or impossible to enter and playgrounds often contain equipment that is not suitable for children with disabilities like SB. Inadequate use or the absence of care facilities are other examples that make it difficult to participate in PA.

#### - Information

Adequate information transfer seems essential, with a variety of informational aspects with several goals. Parents mentioned the scarce attention for and information regarding PA during hospital visits. *“(P) A lot of things you have to find out yourself…I do miss that…..I think, if you’re in a hospital, we visit the hospital regularly, that there should be…..more information…and listening what the child wants and I do miss that……they ask for example ‘how is it’, ‘yes everything goes well’ he (the child) says, well he always says everything goes well……but I think….you should ask ‘what else do you want, how is it going with playing sports, do you play sports’, it is always about what school do you go to and that’s that”*. Information for parents about which sports possibilities are available, specified for what kind of disabilities (mental, physical or a combination) including the care facilities that are available, make it easier to find a suitable sport for the child or adolescent. Several rehabilitation centers and local authorities have sport counselors, which are greatly appreciated because of their individual and practical approach*, “(p) especially one on one, somebody who says…..that’s all available, what kind of child do you have, what kind of situation, where do you live, what are you looking for, leading to something concrete”*. Information for the environment is also emphasized upon, so people understand the possibilities of children and adolescents with SB. Parents themselves sometimes provide this information, but other possibilities are appreciated widely. *“(P) In third grade they spent a lesson on him, they have this book, ….it is about a boy with SB….the teacher read it aloud and then they talked about it”, “(P) we always had support from a regional expertise centre and a therapist comes in ones every so many times”.*

## Discussion

The goal of this study was to describe both personal and environmental factors that are important for participation in physical activity as experienced by both youth with SB and their parents, in order to better develop intervention strategies to improve participation in PA. Three recent reviews looking at PA in persons with SB conclude this is an important gap in current knowledge for this population. They all agree looking into factors that can influence the (maintenance of) PA level is important for the development of interventions to improve PA and fitness levels in a sustainable matter [12,37,38]. In our study, a variety of both positive and negative factors were found on all levels of the PAD-model, both personal and environmental and with varying modifiability. The views of the children and adolescents and the parents were predominantly similar, with only minor differences.

While results are comparable with results presented in two recent systematic reviews about perceived barriers and facilitators to PA for children with disability [18,39], specific factors were associated with the lack of PA in youth with SB. The items specific for youth with SB were mostly related to bowel and bladder care, including assistive devices for these issues, but also the need for privacy in bathrooms or adequate equipment in bathrooms (e.g. changing table) in public places. Another problem specific to children with SB seemed to be a lack of inner drive to initiate any type of behaviour, as reported by parents. This means parents and other adults within the child’s environment need to motivate the child to be active much more than in other children with a disability. Looking at another recent qualitative study, in ambulatory children and adolescents with cerebral palsy, [32]. it shows that themes like ‘contact with and support from people’, ‘mobility and care aids’ and ‘play opportunities’ for example were not found in this study from Verschuren et al. [32]. These differences may be present because our study not only focused on sports participation but on PA in general and also included both ambulating as non-ambulating participants. Another qualitative study by Buffart et al. has looked at factors associated with participation in sports in adolescents and young adults with SB [40]. Results are partly overlapping, while some, like personal goal attainment - e.g. wanting to maintain ambulatory skills (positive)- or having to wake up early (negative) seemed more specific for this older group of patients. Similar though were the lack of information, the limited number of adapted/accessible sports facilities, SB related bowel and bladder complications, equipment issues, fatigue and more general lack of motivation.

Next to negative factors, a wide variety of important positive factors were found in this study. As we know, not all negative factors are modifiable, but may be overcome by using positive factors. The use of assistive devices for optimal mobility and self-care, the development of a sufficient level of fitness, the development of wheelchair skills and self-confidence and a solution-orientated approach were examples of positive factors (both environmental and personal) that contributed to participation in PA. It was very interesting though, to note individual differences that were apparent and the fact that most examples of positive factors were complemented with similar negative factors, meaning the existing positive factors may not be implemented and used enough in general. For example, when children go to regular schools, some physical education (PE) teachers seem to work according to the idea of inclusiveness and find ways to involve children with disability in their lessons, while other teachers do not know how to deal with children with disability. A very active boy for example, walking and exercising with crutches received a fail mark for PE because he could not perform the standard list of required activities (somersault, hopping, etc.).

At the same time though, this study presents a variety of factors for PA and it is evident that the factors vary between the individual children and adolescents. In order to start looking at sustainable interventions to improve participation in PA, these results may serve as a start for developing a practical guide with possible factors contributing to PA that is applicable in individual children and adolescents with SB. Using the contextual factors as represented in the PAD model may help health care professionals to assess the most important factors for the individual child or adolescent in their practice. Currently, we are developing a conversation tool to discuss participation in PA and to identify the factors possibilities or barriers within the (environment) of the individual child. Interventions should be directed at trying to stimulate the positive factors and to deduct the existing negative factors for PA. Even more importantly, health care professionals can work on developing several positive factors . For example, children (and parents) in our study were very enthousiastic about a paralympic athlete, who was a great role model, teaching children to develop skills and confidence through wheelchair training, which was associated with PA lifestyle. If youth encounter positive experiences like this and *know* that they can perform activities, they will feel safer and be confident in performing these activities during daily life. This was also reported by the children. Feeling confident in their wheelchair and being able to negotiate obstacles in daily life (including going up and down the escalator!) gave these children the freedom to be more independent and active in their neighborhood. Considering the results of this study, interventions designed to improve PA should be individualized to the child and multidisciplinary in methods. Physical therapy may be initiated to work on some of the basic requirements to move (e.g. sufficient fitness, certain skills and knowledge regarding an active lifestyle), but they need to work together with other health care professionals if needed for this child, but also teachers at school and coaches at the local sports club. By doing so, the remarks about the scarce information and scarce attention for PA during hospital visits may be overcome, so the future care of these children and adolescents will include concrete actions of preventable consequences of inactivity. It seems important to start early in childhood with promoting independence and the benefits of an active healthy lifestyle. Results of longitudinal studies support the idea that PA in youth is of great importance for the promotion of public health [7] and as the children stated themselves, *“you have to do it yourself later on”*.

The environmental modifiable factors may be partly addressed by health care professionals through advocacy for children with disability and the importance of participation in PA. However, policy makers seem to have a much higher responsibility in dealing with these factors. Several negative environmental factors might be altered by adjustments in policies about health care aspects, but also policies about accessibility and the presence of local sports facilities and possibilities, play possibilities and the attitude of people towards children and adolescents with SB and PA. As cultural context and organization of community based PA varies among countries, the identified factors may differ when interviewing families who live outside of the Netherlands. So it would also be of great interest, if the same research would be conducted in other countries and to see if other factors are being mentioned or if certain negative factors do not exist in other societies as other regulations and standards apply. In the United States for example, the Americans with Disabilities Act [41] is a wide-ranging civil rights law that prohibits discrimination based on disability [42]; this may for example, have an impact on the central theme “accessibility of the environment”. Cultural differences in PA between the Netherlands and the United States have been reported for youth with Cerebral Palsy [43]. If different factors do exist between societies, this might provide insight in strategies that may be used by policy makers to overcome the mentioned negative factors.

Several weaknesses and strengths were present during this qualitative study. Selection bias may be present as only youth and parents might have participated who believed that PA is important. However, both active and inactive youth and their parents participated, as presented in Table 1. At the same time, only Dutch speaking people were included. This could underreport the barriers for PA in non-Dutch speaking ethnic minority groups, as it is known that low levels of health literacy are reported in this population [44,45]. We did not ask about socio-economic status, which given the financial barriers, could have been an important factor. Because the cultural context and organization of community-based physical activity and sports varies among countries, the experiences of the children and parents who participated in this study may differ from those of children and parents who live outside of The Netherlands. Moreover, in this study, the majority of children who participated were non-ambulatory. Thus, it is important to consider that some of the barriers, facilitators, and solutions described in this study might not reflect the experiences of families with children with less severe disabilities. These limitations may of course have influenced the results and the generalizability. At the same time though, a heterogenic group of participants, both ambulatory as non-ambulatory, were included in this study, leading to a wide variety of determinants for PA. Despite this heterogeneity, data saturation was reached and the benefit of this approach was the overall view that could be presented for this population. The final step in our data analysis consisted of categorizing the central themes in modifiable determinants, partly modifiable determinants and non-modifiable determinants. This was done in an effort to reflect on the contextual or personal factors that are present, and may be either positive or negative, but looking at what is a given and not very easy to change or on the opposite factors that could be a goal for intervention. It is certainly true, one could question these labels of (partly) modifiable or not and argue everything is modifiable, but this classification was seen from the perspective of a healthcare provider, working with an individual child. While changes in society are definitely possible, they often require different types of actions, at a societal level.

One of the strengths of this study was using the PAD model in presenting the central themes [31]. By doing so, it was possible to give insight into the different factors of *“Intention”, “Attitude”, “Self-efficacy”, “Health condition”, “Social influence”* and *“Facilitators and Barriers”*. Using the PAD model allows specific and individually tailored interventions to be developed for becoming or maintaining a physically active lifestyle by looking at possibilities within the environmental and personal situation of the child with SB.

Another strength was the general description of PA, not just participation in sports, but also in daily life. This is important, because for most people (non-athletes), PA in daily life is probably a much more important factor in attaining a physically active lifestyle than participation in sports alone. Finally, triangulation, member checking and skeptical peer review were used to meet several important methodological aspects of qualitative research, such as credibility, conformability and dependability [33,34].

## Conclusion

Our findings suggest that while negative factors should be addressed when setting up intervention programs, using positive factors within the individual child seems to be an important starting-point in improving physical activity in youth with SB. Therefore, individual assessment of both personal and environmental factors associated with PA should be standard care within multidisciplinary intervention programs aimed to aimed to encourage healthy active lifestyles in youth with SB.

## Appendix 1 Main topics for the children and adolescents during the focus groups. The topics for the parents were similar, only rephrased.

1. What kind of physical activities do you perform?
2. D you like physical activity and why?
3. What facilitators do you experience regarding physical activity?
4. What barriers do you experience regarding physical activity?
5. What solutions would there be for being more physically active?
